# Retraction: SUMOylation of Mouse p53b by SUMO-1 Promotes Its Pro-Apoptotic Function in Ovarian Granulosa Cells

**DOI:** 10.1371/journal.pone.0248927

**Published:** 2021-03-16

**Authors:** The PLOS ONE Editors

Following the publication of this article [1], concerns were raised regarding the results presented in Fig 1, 3, and 5. Specifically,

- In Fig 1, vertical irregularities have been detected on either side of lane 5 of the SUMO-p53 panel. The authors explained that the underlying blot was spliced to present pertinent results only and provided underlying data confirming the validity of the published panel.
- The β-actin blots of Figs 3A and 3C appear similar. The authors explained that the Fig 3A β-actin blot was inadvertently duplicated, and that as a result the wrong panel was included for the Fig 3C β-actin results.
- In Fig 5A, repetitive clusters of signal have been detected in the FACS results for the HA-p53b and HA-p53b(K375R) panels. The authors provided additional individual level data for the published blots as well as results from triplicate experiments. Assessment of the data deriving from the triplicate experiment samples highlighted similar repetitive clusters of signal between the triplicate results, and the provided FACS results are more similar than would be expected from independent triplicate samples. In addition, the individual level data provided for the published FACS panels shows that for the none, HA, and HA-p53b panels the % Gated results of the upper left quadrants have been reported as the upper right quadrant results and vice versa. The corresponding author clarified that only the lower right quadrants were used to analyse apoptosis rates.

The authors provided some underlying data to support the Figs 1 and 3 results, as well as raw FACS data supporting the results presented in the Fig 5 “none” panel, but the raw data files to support the Fig 5 HA, HA-p53b, and HA-p53b(K375R) results are no longer available.

In light of the concerns affecting the Fig 5A results that question the integrity of the reported FACS data, the *PLOS ONE* Editors retract this article.

LJH agreed with the retraction. XML, FFY, YFY and RZ either did not respond directly or could not be reached.

## References

[1] Liu X-M, Yang F-F, Yuan Y-F, Zhai R, Huo L-J (2013) SUMOylation of Mouse p53b by SUMO-1 Promotes Its Pro-Apoptotic Function in Ovarian Granulosa Cells. PLoS ONE 8(5): e63680. 10.1371/journal.pone.0063680 23696846 PMC3656040

